# Clinical Management and Outcomes of Urosepsis in Relation to Diagnostic Complexity and Microbiological Profile

**DOI:** 10.3390/medicina62050925

**Published:** 2026-05-09

**Authors:** Marcin Talaga, Tomasz Ząbkowski, Kamil Ciechan, Paweł Jędrzejczyk, Tomasz W. Kaminski, Tomasz Syryło

**Affiliations:** 1 Department of General, Functional and Oncological Urology, Military Institute of Medicine—National Research Institute, 128 Szaserów Street, 04-141 Warsaw, Poland; 2 Warsaw Bar Association, 15/16 Żytnia Street, 01-014 Warsaw, Poland; 3 Gen Clinic, 44/U1 Wilhelma Roentgena Street, 02-781 Warsaw, Poland; 4 Hemostasis and Thrombosis Program, Versiti Blood Research Institute, Milwaukee, WI 53226, USA; tkaminski@versiti.org

**Keywords:** urosepsis, diagnostic uncertainty, procalcitonin, multidrug-resistant organisms

## Abstract

*Background and Objectives*: Urosepsis is a common cause of sepsis in adults and is associated with substantial morbidity and mortality, particularly when urinary obstruction delays timely source control. The roles of diagnostic uncertainty at presentation, microbiological phenotypes (including multidrug resistance), and biomarkers in shaping management pathways and outcomes warrant further evaluation. *Materials and Methods*: This retrospective, single-center, observational study included 154 consecutive adult patients hospitalized for urosepsis. Sepsis was defined according to the Sepsis-3 criteria. Baseline clinical modifiers at admission were encoded as binary variables (e.g., malignancy, urinary tract obstruction/altered anatomy, immunocompromised status, acute kidney injury [AKI], and diagnostic uncertainty). Microbiology was standardized into pathogen groups (Gram-negative, Gram-positive, or no isolate), infection complexity (mono- vs. polymicrobial), and multidrug-resistant organism (MDRO) status. Procedures were categorized as no procedure, urinary tract decompression, or other source controls. Biomarkers (C-reactive protein [CRP], procalcitonin [PCT], and creatinine) were analyzed at admission and, when available, during hospitalization. The primary outcomes were in-hospital mortality, ICU admission, and absence/delay of source control. *Results*: The median age was 68 years, and 60.4% of patients were male. The in-hospital mortality and ICU admission rates were 7.1% and 3.9%, respectively. Diagnostic uncertainty was present in 9.8% and was associated with a higher likelihood of no invasive intervention (86.7% vs. 43.9%, *p* = 0.002) and a lower rate of urinary tract decompression (13.3% vs. 45.3%, *p* = 0.01). Gram-negative pathogens predominated (50.0%), and MDROs were identified in 18.2% and were associated with prior urological interventions (53.6% vs. 24.6%, *p* = 0.003) and higher admission PCT levels (8.6 vs. 3.2 ng/mL, *p* = 0.04). Bacteremia was associated with mortality (14.5% vs. 2.2%, odds ratio [OR] 7.64, *p* = 0.007). Mortality was higher in Gram-positive infections (21.7% vs. 4.6%, OR 5.79, *p* = 0.012) and in patients with AKI at admission (25.0% vs. 5.7%; OR, 5.54; *p* = 0.043). *Conclusions*: Urosepsis exhibits distinct clinical and microbiological phenotypes that influence its management and outcomes. Diagnostic uncertainty at presentation was associated with reduced early source control measures, whereas MDRO infections were clustered with prior urological interventions and higher systemic inflammatory burdens. Bacteremia, Gram-positive pathogens, and AKI at admission were associated with an increased in-hospital mortality risk. These findings support a multidimensional early assessment strategy integrating clinical presentation, microbiological risk, biomarkers, and rapid evaluation of obstruction to facilitate timely source control.

## 1. Introduction

Lower urinary tract dysfunction (LUTD) encompasses a heterogeneous group of conditions characterized by abnormalities in urine storage and/or voiding. These disturbances may arise as isolated functional disorders or occur as consequences of neurological impairment, systemic metabolic conditions, traumatic injury, or prior pelvic surgery. Regardless of etiology, LUTD is strongly associated with diminished health-related quality of life, limitations in daily and professional activities, and a heightened risk of urological sequelae, including recurrent urinary tract infections and, in advanced cases, deterioration of upper urinary tract function [1,2].

Overactive bladder is one of the most frequently encountered clinical phenotypes in LUTD. The condition is defined by a compelling need to void that is commonly associated with increased voiding frequency during waking hours and nocturnal episodes, and may occur with or without episodes of urgency-related urinary leakage. These symptoms develop in the absence of urinary tract infection or demonstrable anatomical abnormalities [3]. Contemporary pathophysiological models emphasize the contributions of altered afferent sensory signaling from the bladder, detrusor overactivity, and impaired central modulation of the micturition reflex. These mechanisms may partially account for the suboptimal response to pharmacological therapy observed in some patients [4,5]. In individuals with neurological diseases or injuries, neurogenic LUTD develops due to central or peripheral nervous system dysfunction, with clinical manifestations determined by the level and extent of neural damage. This disorder occurs with increased frequency among individuals affected by multiple sclerosis, spinal cord lesions, and progressive neurological diseases, and is associated with significant consequences for long-term functional status and daily functioning [2,6].

Management of urosepsis is inherently time-dependent and should proceed through parallel pathways, including hemodynamic stabilization, microbiological sampling without unnecessary delay, early initiation of empirical antimicrobial therapy, and active evaluation for obstructive or anatomical factors requiring intervention [7,8,9]. C-reactive protein (CRP), procalcitonin (PCT), and serum creatinine are commonly used biomarkers in the evaluation of patients with suspected urosepsis. CRP reflects a non-specific inflammatory response, whereas PCT is more closely associated with bacterial infection and systemic inflammatory burden. In contrast, serum creatinine serves as a marker of renal function and organ dysfunction, which is a key component of sepsis severity assessment. Together, these biomarkers provide complementary information relevant to diagnosis, risk stratification, and clinical decision-making in urosepsis. Structured algorithm-based approaches in emergency departments may reduce diagnostic ambiguity, shorten the time to source control measures, and ultimately improve clinical outcomes in patients with urinary-source sepsis [7,8,10]. Lower urinary tract dysfunction (LUTD), including overactive bladder and neurogenic bladder disorders, is clinically relevant in the context of urosepsis due to its association with urinary retention, the need for catheterization, and repeated urological instrumentation. These factors may promote bacterial colonization, increase the risk of multidrug-resistant organism (MDRO) infections, and contribute to structural or functional obstruction of the urinary tract. Consequently, patients with LUTD-related conditions may present with increased diagnostic complexity and a higher likelihood of requiring urgent source control interventions, including urinary tract decompression. For this reason, variables reflecting urinary tract obstruction, altered anatomy, and prior urological interventions were incorporated into the present analysis as clinically relevant modifiers.

## 2. Materials and Methods

This study was designed as a retrospective, single-center, observational analysis that included consecutive adult patients hospitalized with urosepsis [11,12]. Complete hospitalization episodes were analyzed, with particular emphasis on the clinical data available at admission and during the early phase of treatment.

Sepsis was defined in accordance with the Sepsis-3 criteria as life-threatening organ dysfunction caused by a dysregulated host response to infection, operationalized as an acute increase in the Sequential Organ Failure Assessment (SOFA) score of ≥2 points or clinically documented organ dysfunction consistent with these criteria.

The diagnosis of urosepsis was established based on the presence of sepsis in conjunction with clinical, laboratory, and microbiological evidence supporting a urinary tract source of infection. This included the presence of urinary tract-related symptoms or signs, positive microbiological cultures from urine, blood, or upper urinary tract specimens (e.g., percutaneous nephrostomy), and the absence of an alternative identifiable source of infection based on clinical evaluation and available diagnostic data.

To minimize the risk of misclassification, cases with bacteremia of unclear origin and incidental bacteriuria without supportive clinical or laboratory findings were not classified as urosepsis. In cases with incomplete data, source attribution was based on the overall clinical context documented in the medical records.

Adult patients admitted for urosepsis were eligible for inclusion [11,12]. Most admissions occurred in the emergency setting. The study cohort predominantly comprised older adults [12]. No additional exclusion criteria were applied apart from the availability of complete clinical documentation required for the analysis of predefined variables.

Sepsis was defined according to the Sepsis-3 criteria as life-threatening organ dysfunction due to infection, operationalized as an acute increase in the Sequential Organ Failure Assessment (SOFA) score of ≥2 points or clinically documented organ dysfunction consistent with these criteria.

Urosepsis was defined as sepsis with a clinically supported urinary tract source, based on urinary symptoms or signs, positive microbiological findings (urine, blood, or upper urinary tract specimens), and exclusion of an alternative infection focus. Cases with bacteremia of unclear origin and incidental bacteriuria without supporting evidence were not classified as urosepsis.

“Diagnostic uncertainty” was defined as explicit documentation in admission or emergency records indicating an unclear infection source or atypical presentation and was assessed at initial evaluation as a binary variable based on predefined criteria.

Clinical variables were extracted from the medical records and encoded as binary indicators based on explicit documentation. These include active malignancy, urinary tract obstruction or altered urological anatomy, immunocompromised status, acute kidney injury (AKI) at presentation, and diagnostic uncertainty at the emergency department level [7,11,12]. Narrative clinical notes were used exclusively to identify clearly documented modifiers and were not directly incorporated into statistical analyses. Acute kidney injury (AKI) at admission was defined according to KDIGO criteria as an increase in serum creatinine of ≥0.3 mg/dL within 48 h or ≥1.5 times the baseline value. Baseline creatinine was defined as the most recent pre-admission value; if unavailable, admission creatinine was used as reference. Urine output criteria were not included due to incomplete data.

Bacterial infection was defined based on the microbiological results obtained during hospitalization, including cultures from blood, urine, and material collected from percutaneous nephrostomies (PCNs) [7,12]. Cultures from drains or surgical wounds were included in the selected cases. All microbiological data were initially extracted in a narrative format and subsequently standardized for statistical analysis.

Pathogens were classified into simplified, clinically relevant categories using the variable *Pathogen_group*, comprising Gram-negative bacteria, Gram-positive bacteria, or no isolates [12]. The spectrum of isolated Gram-negative pathogens comprised *Escherichia coli*, *Klebsiella* species, *Proteus* species, *Pseudomonas aeruginosa*, as well as representatives of the *Enterobacter*, *Citrobacter*, and *Morganella* genera. In contrast, Gram-positive isolates were primarily identified as *Enterococcus* species and *Staphylococcus* species [7,12].

Multidrug-resistant organisms (MDROs) [13] were defined in accordance with internationally accepted criteria as acquired non-susceptibility to at least one agent in three or more antimicrobial categories. Antimicrobial susceptibility testing was interpreted according to European Committee on Antimicrobial Susceptibility Testing (EUCAST) standards [14].

Microbiological findings were interpreted in conjunction with clinical presentation and source of sampling to distinguish clinically relevant infection from possible colonization, particularly in non-sterile specimens [7,12].

Infection complexity was categorized as monomicrobial or polymicrobial based on the number of isolated pathogens [12]. Rare or atypical organisms and mixed flora were exclusively recorded for supplementary analysis.

Information regarding urological and surgical interventions performed during hospitalization was initially recorded descriptively and subsequently classified into *Procedure_main*, which comprised three categories: no procedure (conservative management), urinary tract decompression, and other source-control procedures [7,8,12]. Urinary tract decompression was stratified according to anatomical level into upper-tract decompression (percutaneous nephrostomy or ureteral stenting) and lower-tract drainage (e.g., suprapubic or transurethral catheterization). These interventions were analyzed separately due to their differing clinical indications and relevance in obstructive urosepsis.

Due to the retrospective nature of the study and incomplete time-resolved data, detailed analysis of time-to-decompression was not feasible.

A separate binary variable (*Prior_urologic_intervention*) identified patients who underwent urological or surgical procedures immediately before hospitalization, potentially contributing to the development of sepsis [7,12].

Laboratory assessment encompassed selected inflammatory markers and indicators of renal function, specifically measurements of C-reactive protein (CRP), procalcitonin (PCT), and serum creatinine [8,11]. The values obtained at admission were analyzed for all patients, and serial measurements during hospitalization were evaluated to assess temporal trends.

The main study endpoints comprised in-hospital death, requirement for admission to an intensive care unit (ICU), and failure to implement, or postponement of, definitive source control interventions during the hospital stay [8,11,12]. Source control was defined as any intervention aimed at eliminating the infectious focus, including upper urinary tract decompression (percutaneous nephrostomy or ureteral stenting), bladder drainage, or other indicated procedures.

Delay in source control was defined as failure to perform an indicated intervention within 24 h of hospital admission, while absence referred to cases in which no such intervention was performed despite clinical indication.

Statistical analyses were performed using nonparametric methods appropriate for data distribution. Relationships between qualitative variables were assessed with either Fisher’s exact test or the chi-square test, depending on data distribution. Differences in quantitative measures were analyzed using the Mann–Whitney U test. Odds ratios (ORs) and corresponding confidence intervals were calculated for the selected outcomes. Correlations were assessed using Spearman’s rank correlation coefficients. Statistical significance was defined as a two-sided *p*-value < 0.05. All reported odds ratios are unadjusted and should be interpreted as exploratory associations rather than causal effects.

## 3. Results

The analysis included 154 consecutive adult patients hospitalized with urosepsis. Most admissions were acute (91.6%), with only a small proportion of elective admissions (7.8%). The cohort was largely composed of elderly individuals, with a median age of 68 years (spanning from 26 to 99 years), and demonstrated a modest predominance of men, who accounted for 60.4% of the study population.

At presentation, active malignancy was documented in 20.3% of the patients, whereas urological obstruction or altered urinary tract anatomy was observed in 10.5%. AKI at admission was identified in 7.8%, whereas a documented immunocompromised status was uncommon (1.3%).

Overall, in-hospital mortality was 7.1% (11/154), and 3.9% of patients required ICU admission.

Diagnostic uncertainty at the emergency department level was identified in 15 patients (9.8%), whereas the remaining 90.2% presented a clinically clear uroseptic picture. Patients with diagnostic uncertainty had higher rates of active malignancy and AKI at presentation; however, these differences were not statistically significant.

Diagnostic uncertainty is strongly associated with differences in management strategies. Patients with an uncertain presentation were significantly more likely to receive no urological intervention during hospitalization (86.7% vs. 43.9%, *p* = 0.002) and were significantly less likely to undergo urinary tract decompression (13.3% vs. 45.3%, *p* = 0.01).

Microbiological results revealed that infections were most commonly caused by Gram-negative organisms (50.0%), followed by Gram-positive pathogens (14.9%). In 35.1% of cases, no pathogens were isolated.

Most infections were monomicrobial (67.5%), while polymicrobial infections were rare (1.9%). In 30.5% of cases, infection complexity could not be definitively classified owing to incomplete microbiological data. Infection complexity could not be classified in 30.5% of cases due to incomplete microbiological data. Sensitivity analysis restricted to cases with complete data showed consistent results.

MDROs were identified in 18.2% (28/154) of patients. MDRO infections were significantly more often caused by Gram-negative bacteria (75.0% vs. 44.4%, *p* = 0.004) and were more frequently monomicrobial (85.7% vs. 63.5%, *p* = 0.02).

Blood cultures were positive in 40.3% of the patients, urine cultures were positive in 16.2%, and cultures obtained from PCNs were positive in 7.1%. Other sources accounted for 36.4% of the sample. Microbiological source categories were defined based on the primary site of pathogen isolation used for analytical classification, rather than all collected specimens. In cases where multiple samples were obtained, classification was based on the clinically most relevant or first positive isolate.

Blood cultures were obtained in the majority of patients, with a positivity rate of 40.3%, while urine cultures and percutaneous nephrostomy (PCN) cultures showed lower positivity rates. Multiple sampling sites were frequently used in clinical practice; however, the reported distribution reflects the primary microbiological classification rather than cumulative sampling. The relatively high proportion of cases without identified pathogens (35.1%) likely reflects both prior antibiotic exposure and limitations of routine clinical sampling.

Isolation of pathogens from the blood was strongly associated with mortality (14.5% vs. 2.2%, OR = 7.64, *p* = 0.007), indicating a substantially higher risk of adverse outcomes in bacteremic patients.

Nearly half of the cohort (48.1%) did not undergo invasive procedures during hospitalization. Urinary tract decompression was performed in 42.2%, while other source control procedures were performed in 9.7%.

A history of prior urological interventions before admission was documented in 29.9% of patients.

Patients with MDRO infections were also more likely to require urinary tract decompression during hospitalization (60.7% vs. 38.1%, *p* = 0.03).

**Figure 1:**
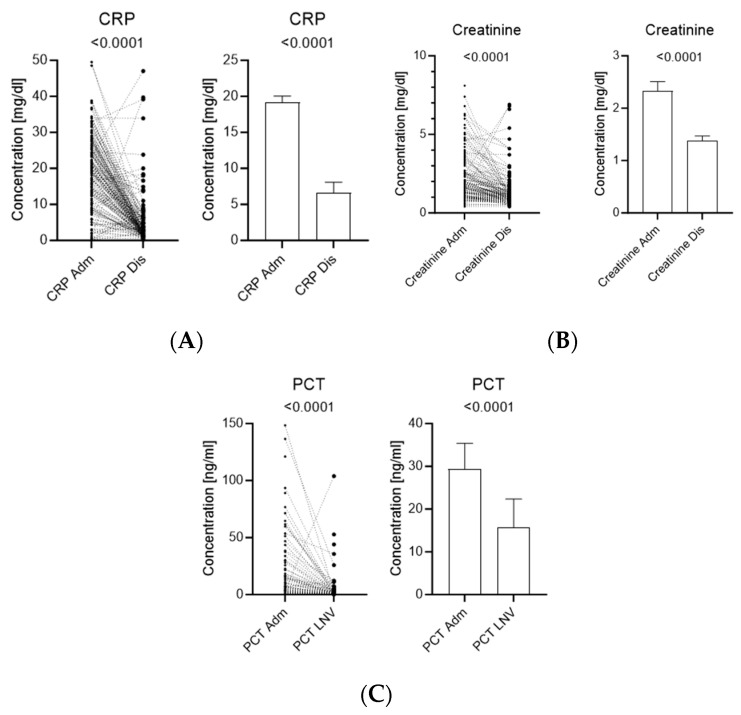
Changes in biomarker levels between hospital admission and discharge in patients with urosepsis. Paired comparisons of serum creatinine (**A**), C-reactive protein (CRP) (**B**), and procalcitonin (PCT) (**C**) measured at hospital admission and discharge. Individual patient values are shown as paired points connected by lines to illustrate within-patient changes over time, with summary bars indicating mean values ± SE. Analyses were restricted to patients with available measurements at both time points (paired dataset; n varies per biomarker—Creatinine n = 154, CRP n = 154, and PCT n = 107). Missing data were not imputed. Statistical significance was assessed using the paired *t*-test, and corresponding *p*-values are indicated in each panel.

At admission, the median serum creatinine level was 1.65 mg/dL (interquartile range [IQR] 1.00–2.60), which decreased to 1.00 mg/dL (IQR 0.80–1.50) at discharge. Median CRP levels declined from 19.4 mg/dL (IQR 11.5–25.9) at admission to 2.50 mg/dL (IQR 1.30–4.85) at discharge (Figure 1).

The median PCT level at admission was 3.90 ng/mL (IQR 0.86–18.4). Patients with MDRO infections had significantly higher PCT levels than those without MDRO infections (8.6 vs. 3.2 ng/mL, *p* = 0.04) (Figure 1). Trends toward higher CRP and creatinine levels were also observed in the MDRO group.

Mortality rates were significantly higher in patients with Gram-positive infections (21.7% vs. 4.6%, OR = 5.79, *p* = 0.012) and AKI at presentation (25.0% vs. 5.7%, OR = 5.54, *p* = 0.043).

Infections caused by multidrug-resistant organisms were linked to increased rates of in-hospital death and intensive care unit admission, although the observed differences did not reach statistical significance.

**Figure 2:**
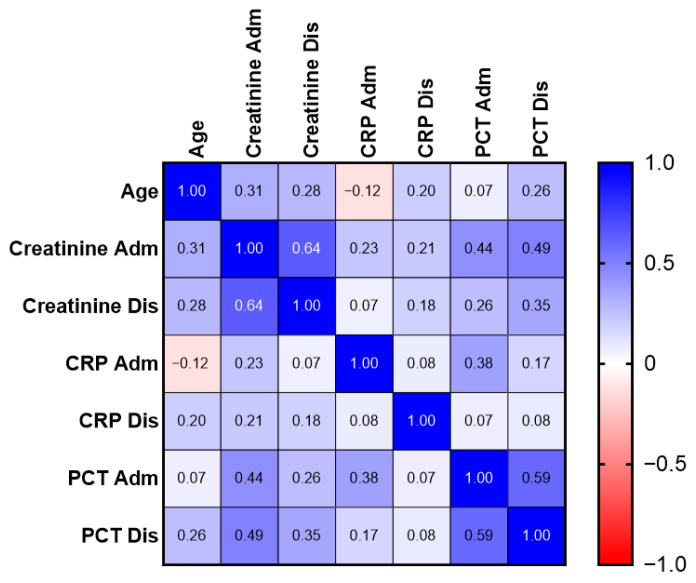
Spearman correlation matrix of demographic and laboratory parameters in patients with urosepsis. Spearman’s rank correlation heatmap illustrating associations between age and laboratory parameters measured at hospital admission (Adm) and discharge (Dis), including serum creatinine, C-reactive protein (CRP), and procalcitonin (PCT). Correlation coefficients (ρ) are displayed within each cell, with color intensity indicating the strength and direction of the association (blue = positive correlation; red = negative correlation). The analysis was performed using pairwise complete observations (available-case analysis), with missing data handled by excluding incomplete pairs for each correlation. All correlations are based on nonparametric Spearman rank testing.

**Figure 3:**
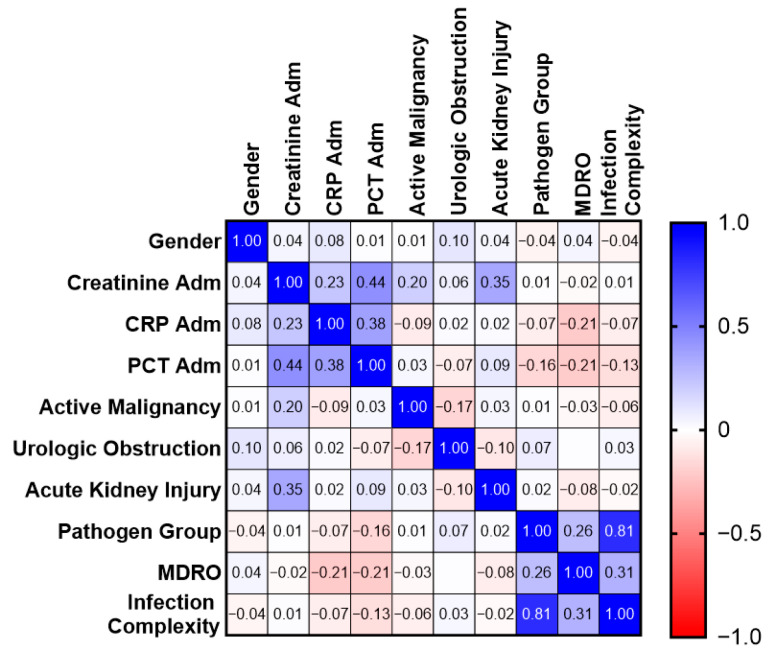
Spearman correlation matrix of clinical characteristics and infection-related variables in patients with urosepsis. Spearman’s rank correlation heatmap showing associations between demographic variables (gender), laboratory parameters at hospital admission (serum creatinine, C-reactive protein [CRP], and procalcitonin [PCT]), comorbid conditions (active malignancy, urological obstruction, and acute kidney injury), and infection-related characteristics (pathogen group, multidrug-resistant organism [MDRO] status, and infection complexity). Correlation coefficients (ρ) are displayed within each cell, with color intensity representing the strength and direction of the association (blue = positive correlation; red = negative correlation). The analysis was performed using pairwise complete observations (available-case analysis), with missing data handled by excluding incomplete variable pairs.

Spearman’s correlation analysis demonstrated a strong correlation between renal function parameters at admission and discharge (R = 0.64, *p* < 0.001). Admission PCT levels moderately correlated with serum creatinine levels (R = 0.44, *p* < 0.001), suggesting that PCT may reflect both systemic inflammatory burden and renal dysfunction (Figure 2). Notably, moderate positive correlations were observed between pathogen group and infection complexity (ρ = 0.81), and between MDRO status and infection complexity (ρ = 0.31), while weaker associations were present between inflammatory markers and clinical variables (Figure 3).

Diagnostic uncertainty showed a significant negative correlation with the likelihood of undergoing surgical or urological intervention (R = −0.48, *p* < 0.001). The MDRO status correlated with the pathogen group and infection complexity, confirming a coherent microbiological phenotype.

## 4. Discussion

This retrospective observational analysis indicates that the progression of urosepsis is influenced by a complex interplay of patient-related factors present at baseline, pathogen characteristics, and the timeliness and adequacy of source-control interventions. Our findings support the concept that urosepsis represents a heterogeneous clinical syndrome rather than a uniform entity, with distinct phenotypes associated with different risk profiles and outcomes [11,12,15].

A key observation of this study was the impact of diagnostic uncertainty at the emergency department level on subsequent management. Patients with atypical or non-specific initial presentations were significantly less likely to undergo early urological source control measures and more frequently received conservative treatment alone. This finding is clinically relevant and aligns with previous reports indicating that urosepsis—particularly in older adult patients or those with malignancy or organ dysfunction—may present with extra-urological symptoms, delaying recognition of the “infection plus obstruction” scenario [12,16,17]. Current recommendations from the European Association of Urology and the Surviving Sepsis Campaign emphasize that postponement of effective source control is a critical factor associated with unfavorable outcomes in sepsis, regardless of the early initiation of appropriate antimicrobial treatment [18].

MDRO infections constituted a distinct and clinically coherent phenotype in our cohort. MDRO urosepsis is significantly associated with prior urological interventions, altered urinary tract anatomy or obstruction, and a predominance of Gram-negative pathogens. These associations are biologically plausible and consistent with existing literature identifying prior instrumentation, indwelling devices, and upper urinary tract involvement as major drivers of resistant infections [12,15,18]. Importantly, MDRO infections were also characterized by higher PCT levels on admission, suggesting a greater systemic inflammatory burden and potentially more severe disease.

Our biomarker analysis further supports the role of procalcitonin as a clinically informative marker of urosepsis. Compared to CRP, PCT levels showed stronger associations with renal dysfunction, MDRO phenotype, and markers of disease severity. These results align with current data suggesting that procalcitonin is a more sensitive indicator of bacterial burden and systemic inflammatory response, especially in cases of bacteremia and severe urinary tract infections [8,19]. Importantly, this relationship should be interpreted with caution, as PCT also showed a moderate correlation with serum creatinine, indicating potential confounding by renal dysfunction. Elevated PCT levels in these settings may reflect both increased inflammatory burden and impaired renal clearance, particularly in patients with acute kidney injury [20,21]. Nevertheless, biomarker elevation alone should not be interpreted as a surrogate for source control decisions. Rather, it should complement clinical assessments and imaging findings [15].

Notably, bacteremia, Gram-positive pathogens, and AKI at presentation emerged as strong predictors of in-hospital mortality in this cohort. The association between Gram-positive bacteremia and worse outcomes has been less frequently emphasized in urosepsis but may reflect a subset of patients with advanced systemic involvement or healthcare-associated infections. Similarly, AKI upon admission remains a robust marker of poor prognosis, reinforcing the importance of early organ dysfunction in sepsis risk stratification [10,11].

Collectively, our findings highlight the clinical value of an integrated algorithm-based approach for suspected urosepsis. Early parallel assessment, including clinical evaluation, microbiological sampling, biomarker measurement, and rapid imaging for obstruction, may reduce diagnostic uncertainty and facilitate timely source control. Such an approach has direct implications for emergency care pathways and antimicrobial stewardship, particularly in populations at a high risk of resistant infections or atypical presentations [15,17].

Several limitations should be acknowledged. The retrospective nature and single-institution setting restrict the ability to draw causal conclusions and limit external validity. The modest cohort size, especially within stratified analyses, may have constrained the power to identify weaker associations. Moreover, gaps in microbiological reporting and incomplete biomarker measurements, which reflect routine clinical conditions, could have contributed to information bias. Additionally, kidney-function-stratified analyses were not feasible due to sample size constraints, and therefore conclusions regarding the independent prognostic value of PCT should be considered exploratory.

Despite these limitations, the internal consistency of the observed associations and their concordance with the existing literature support the validity of our conclusions.

## 5. Conclusions

This retrospective, single-center observational study demonstrated that the clinical course and outcomes of urosepsis are determined by the combined effects of baseline patient status, microbiological characteristics, and the timeliness of source control measures.

First, diagnostic uncertainty at hospital admission was associated with a lower likelihood of early source control measures. Patients presenting with nonspecific or atypical clinical features were less frequently subjected to prompt urological intervention, suggesting that the delayed recognition of obstructive urosepsis may contribute to suboptimal management.

In addition, infections attributable to multidrug-resistant organisms appear to constitute a separate clinical subgroup. Urosepsis related to MDROs occurred more often in individuals with prior urological procedures and was characterized by a more pronounced inflammatory response, as indicated by higher procalcitonin concentrations. This pattern suggests that MDRO-associated infections are linked to specific patient- and procedure-related risk factors rather than arising randomly.

Furthermore, the occurrence of bloodstream infection and the involvement of Gram-positive bacteria were both linked to increased in-hospital mortality, emphasizing the prognostic significance of systemic spread and microbial characteristics in urosepsis. Acute kidney injury at presentation also emerged as a strong predictor of unfavorable outcomes, underscoring the value of early evaluation of renal function for effective clinical risk stratification.

Fourth, among the evaluated biomarkers, PCT demonstrated a stronger association with disease severity and an unfavorable clinical course than conventional inflammatory markers, such as CRP. Nevertheless, biomarker assessment should be regarded as an adjunct to, rather than a substitute for, timely clinical decision-making and source control.

Overall, the findings support the need for an integrated, multidimensional approach for the early assessment of patients with suspected urosepsis. Incorporating clinical presentation, microbiological risk factors, biomarker profiles, and evaluation of urinary tract obstruction may facilitate earlier source control measures and improve clinical outcomes.

These findings may inform the development of structured emergency department algorithms for suspected urosepsis, particularly in patients with atypical presentations or prior urological interventions.

## Data Availability

The data supporting the findings of this study are available from the corresponding author upon reasonable request. During the preparation of this manuscript, the authors used ChatGPT (OpenAI, GPT-5.4 Thinking; accessed on submitted manuscript) to assist with abstract shortening, literature research, and shortening of selected sentences to improve linguistic clarity. The authors have re-viewed and edited the output and take full responsibility for the content of this publication.

## Abbreviations

The following abbreviations are used in this manuscript:

LUTD	Lower urinary tract dysfunction
UTI	Urinary tract infection
AKI	Acute kidney injury
PCN	Percutaneous nephrostomy
MDRO	Multidrug-resistant organism
CRP	C-reactive protein
PCT	Procalcitonin
ICU	Intensive care unit
OR	Odds ratio
IQR	Interquartile range
